# Intramedullary Spinal Cord Metastasis from Renal Cell Carcinoma: A Systematic Review of the Literature

**DOI:** 10.1155/2018/7485020

**Published:** 2018-12-16

**Authors:** Yuxiang Weng, Renya Zhan, Jian Shen, Jianwei Pan, Hao Jiang, Kaiyuan Huang, Kangli Xu, Hongguang Huang

**Affiliations:** Department of Neurosurgery, The First Affiliated Hospital, School of Medicine, Zhejiang University, Hangzhou, China

## Abstract

Intramedullary spinal cord metastases from renal cell carcinomas (RCCs) are rare and can cause serious diagnostic and therapeutic dilemmas. The related reports are very few. This review was aimed to perform an analysis of all reported cases with intramedullary spinal cord metastases from RCCs. In January 2018, we performed a literature search in PubMed database using a combination of the keywords “intramedullary spinal cord metastasis” and “renal cell carcinoma”. In addition, we present the clinical, neuroradiological, and histopathological findings in our patient with an intramedullary metastasis from a RCC. 17 cases were generated in our research. The mean interval from diagnosis of RCC to diagnosis of ISCM was 22 months. The median survival of surgically treated patients was 8.6 months and 8 months in patients who underwent radical surgery. Based on our review, RCCs can invade the medulla of the spinal cord several years after removal of the primary lesion. The prognosis of ISCMs from RCCs was poor. Retrograde passage of tumor cells into the spinal cord from the inferior vena cava via the epidural venous sinuses may have been the pathological mechanism for ISCM in our patient. Radical resection and radiation are effective ways of achieving recovery of neurologic function and improving quality of life. More reports are needed to enable exploration of the mechanisms of metastasis and the optimal forms of therapy.

## 1. Introduction

Intramedullary spinal cord metastases (ISCMs) are rare. They mostly originate from lung and breast cancers but can originate from a wide variety of other solid tumors, including melanoma, malignant lymphoma, and colon, ovarian, and renal cell carcinomas (RCCs) [1]. ISCMs are generally associated with poor survival. Because they are rare and there are no pathognomonic symptoms, diagnosis is often unduly delayed [2]. Recommended treatments include surgery and radiotherapy; however, there is no consensus on optimal treatment. Heightened awareness of this entity may lead to earlier diagnosis and thus treatment at a stage when neurological deficits may be reversible and more effective palliation may be achieved. There are only a few published reports of ISCMs from RCCs. We here provide an overview of clinical data about this rare entity.

## 2. Methods

In January 2018, we performed a literature search which reported the patient with intramedullary spinal cord metastases from renal cell carcinoma in PubMed database using a combination of the keywords “intramedullary spinal cord metastasis” and “renal cell carcinoma”.

## 3. Results

### 3.1. Our Case

**Figure 1:**
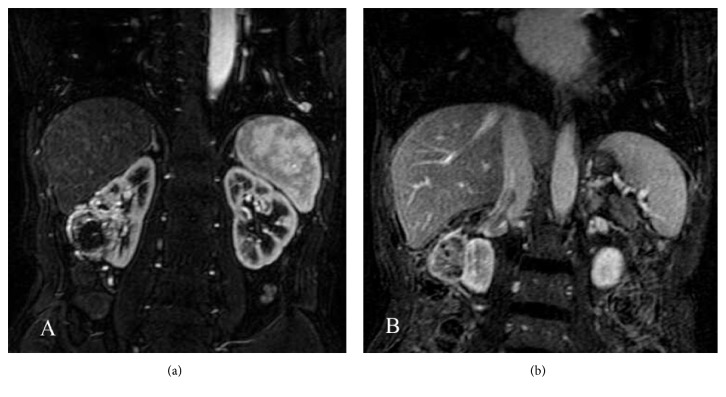
(a) Abdominal MRI demonstrating right renal cell carcinoma (arrow). (b) MRI also demonstrating tumor thrombosis in the inferior vena cava.

A 58-year-old man presented with numbness and aching pain of both lower extremities without weakness that had been progressive over the previous 2 months and development of paraparesis 3 days before presentation. Physical examination revealed muscle strength of 0/5 below the knee with absence of sensation and 3/5 for the proximal lower limbs. He gave a history of a right RCC with lung metastasis approximately 3 years previously. The renal mass had been discovered by MRI, which had also shown tumor thrombosis in both the renal vein and inferior vena cava (Figures 1(a)-1(b)). The patient had undergone right radical nephrectomy and inferior vena cava dissection with embolectomy and the diagnosis of RCC had been confirmed pathologically. He had also received treatment with interlukin-2 and interferon postoperatively.

**Figure 2:**
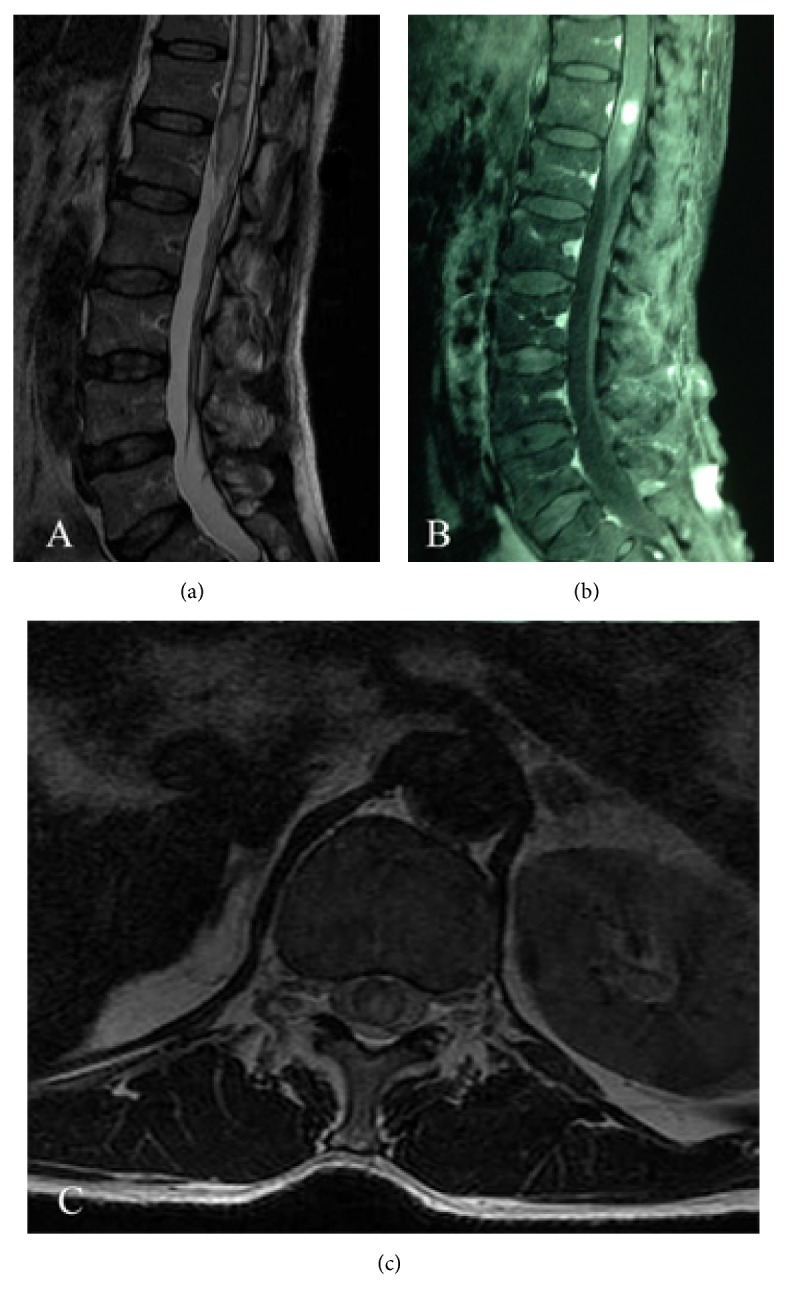
(a) Preoperative T2-weighted sagittal MRI demonstrating increased signal intensity. (b) Preoperative T1-weighted sagittal MRI revealing a well-defined tumor with significant enhancement after gadolinium injection. (c) Preoperative T1-weighted axial MRI showed well-circumscribed lesion inside the spinal cord at the T12 level.

**Figure 3:**
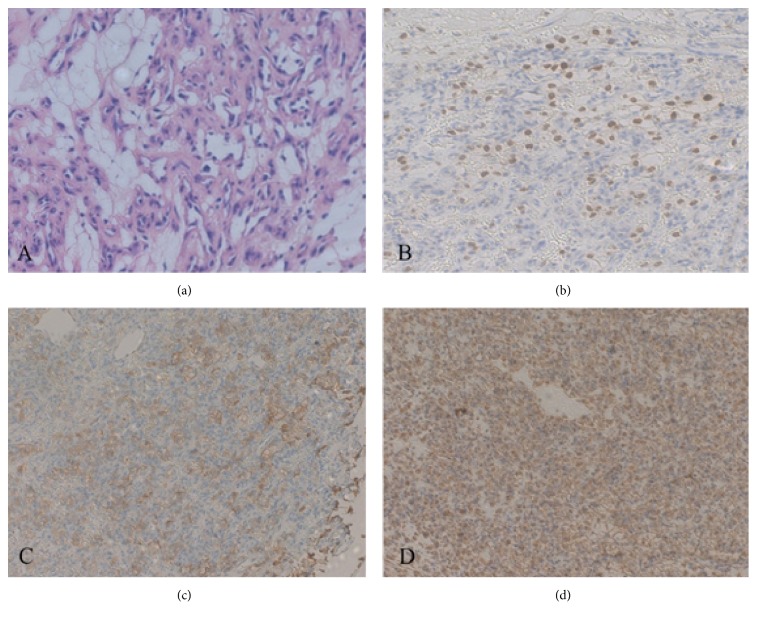
(a) Photomicrograph demonstrating clear tumor cells. Hematoxylin and eosin, original magnification × 100. Immunohistochemical staining for (b) PAX8, (c) CK(pan), and (d) vimentin showing strong cytoplasmic reactions within tumor cells. Original magnification all × 100.

**Figure 4:**
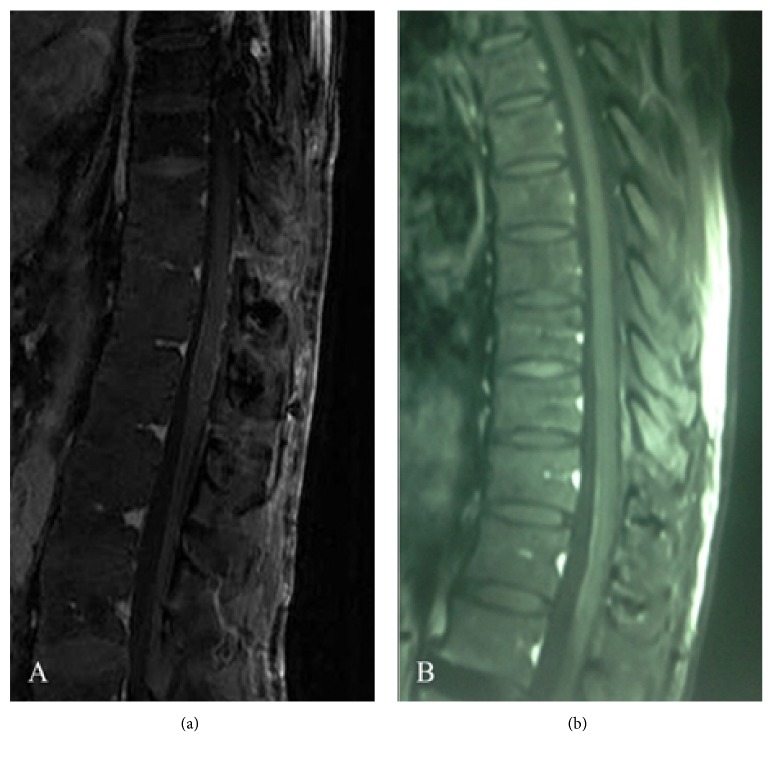
(a) T1-weighted sagittal MRI after surgery and (b) at a 6-month follow-up showing no evidence of a mass.

Magnetic resonance imaging (MRI) of the spine demonstrated a 15 mm × 7 mm intramedullary mass at the T12 level with high signals on T2WI, hypointense signals on T1WI, and significant enhancement with intravenous gadolinium (Figures 2(a)–2(c)). Considering the history of RCC and rapid progression of his neurological deficit, the provisional diagnosis was ISCM. The patient underwent a T11 and T12 laminectomy during which the spinal dura mater was opened through a midline posterior sulcus incision under magnification and a tumor identified at the T12 level. After gentle and careful dissection, the lesion was completely removed. The pathological diagnosis was RCC. Immunochemical staining demonstrated the following: CD10(+), paired box protein 8 (PAX8)(+), creatine kinase (CK) (pan)(+), vimentin(+), and P504S(+)(Figures 3(a)–3(d)). A postoperative MRI confirmed complete removal of the lesion (Figure 4(a)). The muscle power improved to 3/5 below the knee in both lower limbs and sensation recovered partially. The patient received a course of focal radiation therapy. MRI of the spinal cord revealed no recurrence at a 6-month follow-up (Figure 4(b)).

### 3.2. Patients Characteristics

The characteristics of reported cases with ISCMs that originated from RCCs, including our patient, are presented in Table 1 [3–13]. The average age of presentation is 51 (37–70) years; 65% of reported patients were male and 35% female. Cervical localization was reported in 41%, thoracic in 41%, and lumbar in 18% of patients. Five of 17 reported patients with ISCMs from RCCs had left-sided primary tumors, eight right-sided, and two bilateral; the localization of the remaining two was not reported. Weakness was present at diagnosis of ISCM from RCC in 71% of patients. Backache (41%), dysesthesia (41%), and sphincter dysfunction (35%) were also common. Radicular pain and Brown-Séquard syndrome were each seen in 18%. Most patients were diagnosed by MRI (94%), the exception being one by myelography. Contrast enhancement was seen in 92% of patients to whom gadolinium was administered. The mean interval from diagnosis of RCC to diagnosis of ISCM was 22 months (0 months–15 years). Lung (59%) and brain (47%) were the most common sites of simultaneous metastases. Bone (n=3), liver (n=1), contralateral kidney (n=1), adrenal gland (n=1), and systemic organ metastasis (n=1) were the other reported sites. Conservative therapy was selected in 37.5% of cases and surgery in 62.5%. The median survival of surgically treated patients was 8.6 months (n=10) and 8 months in patients who underwent radical surgery (n=4).

## 4. Discussion

ISCMs are rare, accounting for 0.1% to 2% of all spinal cord tumors [14]. Lung and breast cancer are the commonest sources of ISCMs, with RCCs accounting for only 4%–9% of such tumors [1, 15]. According to our review, eleven of the patients we reviewed developed ISCM within one year of diagnosis of RCC; in five patients the ISCM was present at the time of diagnosis of RCC [6, 9, 12, 13]. Five patients developed metastases a year or more after diagnosis of RCC occurrence [3, 6–8]; all had undergone nephrectomy similarly to our patient whose metastasis was diagnosed 34 months after nephrectomy. These data suggest that nephrectomy may delay ISCMs from RCCs.

Three possible pathological mechanisms have been suggested for how RCCs metastasize to the spinal cord parenchyma [1]. The most common mechanism is likely hematogenous spread via one of two pathways. One is an arterial route, which is supported by coexistence of lung and brain metastases. Another hematogenous route is through the vertebral venous plexus (Batson's venous plexus), which extends from the pelvis to the cranial venous sinuses, enabling retrograde transportation to the spinal cord. The second mechanism is associated with meningeal carcinomatosis. Tumor cells from carcinomatous meningitis may infiltrate blood vessels, penetrate the spinal cord, and finally invade the spinal cord parenchyma. The third mechanism is direct invasion through invasion of metastatic tumor cells from the spinal extradural space, cerebrospinal fluid, or nerve roots. As Kamerath et al. [16] have reported, absence or thrombosis of the inferior vena cava can cause epidural venous plexus enlargement. Moreover, the epidural plexus system has no valves, allowing considerable collateral flow. Therefore, it is possible for tumor cells to travel in a retrograde fashion into the spinal cord from a vessel lumen. Our patient had extensive invasion of the inferior vena cava and renal vein with formation of tumor thrombus, the lung being the only site of systemic metastases, at the time of diagnosis of his RCC. However, the ISCM from his RCC manifested 3 years after nephrectomy. Moreover, there was a spatial relationship in that his ISCM was located at the same level as the previous RCC. We consider this potent evidence that tumor cells travelled in a retrograde fashion into the spinal cord from the inferior vena cava via the epidural venous sinuses.

ISCMs have a poor prognosis. Germ et al. [17] found that more than 80% of patients died within 3 months. Treatment options for ISCM include radiation with or without steroid therapy, surgery, chemotherapy, immunotherapy, and targeted therapy. Because of the blood–brain barrier, chemotherapy is regarded as inappropriate for ISCMs [9]. Immunotherapy such as interferon-alpha and interleukin-2 lacks efficacy and produces serious adverse reactions; further clinical trials are needed [18, 19]. Some research showed targeted drugs could improve the outcomes of patients with metastatic RCC. The monoclonal antibody against vascular endothelial growth factor, mammalian target of rapamycin inhibitor, and the multitargeted tyrosine kinase inhibitor are first-line options [20]. However, the use of targeted drugs in patients with ISCMs from RCCs is lack of sufficient research. Although RCCs are radioresistant tumors, radiation may achieve longer survival times: Schiff et al. [21] reported that the median survival of patients who received radiotherapy was 4 months compared with 2 months for those who did not receive radiotherapy. Our review of published reports yielded a mean survival time after radiotherapy of 8 months [3–13]. Moreover, radiotherapy often results in improvement in neurological deficits [6, 9, 11]. The patient reported by Parikh et al. [9] is particularly worth mentioning: their patient with ISCM from RCC was still alive with good neurologic status 26 months after fractionated stereotactic radiosurgery preceded by treatment by external beam radiotherapy for progressive neurologic symptoms. What is more, stereotactic radiosurgery has been administered to ISCM from other primary tumors and appears to be an effective and safe method of treating such patients [22–24], suggesting that fractionated stereotactic radiosurgery is an effective modality for treating ISCMs from RCCs. With developments in surgical techniques and equipment, doctors are increasingly selecting surgery as their first choice. Actually all recently reported patients with ISCMs from RCCs who underwent microsurgery had good recovery of neurologic function [9, 12, 15]. Kalayci et al. [1] reviewed 138 patients with ISCMs and reported that survival was nearly twice as long in patients who underwent resection compared to that in those who were treated conservatively. Interestingly, Park et al. [10] reported a patient with an ISCM from RCC who underwent emergency surgery because of rapid progression of paraparesis during focal radiotherapy; this patient's motor power improved enough for recovery of ambulation. Therefore, surgery is an important modality for salvage of patients with rapidly progressing neurological deficits, especially those whose ISCMs originated from radioresistant tumors such as RCCs. Additionally, Payer et al. [25] reviewed 22 patients who had undergone surgery for ISCMs over a 22-year period and commented that surgery can now be performed with minimal new morbidity and results in maintenance of neurological performance status. To our knowledge, no studies have compared these two treatment modalities to determine which is better. Our patient's rapidly deteriorating neurological function prompted us to perform surgery and adjuvant radiotherapy after resection, his symptoms improved, and no masses were detected in his spinal cord at a six-month follow-up. Thus, our results may indicate that radical resection should be the first choice for patients with ISCMs who have rapidly progressive neurological deficits.

## 5. Conclusions

RCCs can invade the medulla of the spinal cord several years after removal of the primary lesion. Retrograde passage of tumor cells into the spinal cord from the inferior vena cava via the epidural venous sinuses may have been the pathological mechanism for ISCM in our patient. Radical resection and radiation are effective ways of achieving recovery of neurologic function and improving quality of life. More reports are needed to enable exploration of the mechanisms of metastasis and the optimal forms of therapy.

## Figures and Tables

**Table 1:** Case reports of ISCM from RCC.

Author	Age/ sex	Location	RCC location	Diagnosis	Contrast enhancement of MRI	Time from diagnosis of RCC to ISCM (months)	Other RCC metastasis	Treatment	Outcome	Survival after diagnosis of ISCM (months)
Schijns et al.	70/F	C7	left	MRI	yes	0	liver, contralateral kidney	surgery	improved	>12m
Poggi et al.	37/M	T12	right	MRI, PET	yes	3	lung, bone, brain	radiation	unknown	unknown
Fakih et al.	56/M	C4	right	MRI	unknown	0	lung, brain	radiation	improved	6m
Fakih et al.	60/M	T2	right	MRI	unknown	180	lung, brain	surgery+radiation	improved	5m
Fakih et al.	68/F	L1	-	myelography	-	2	-	radiation	improved	16m
Fakih et al.	57/F	C7	-	MRI	yes	0	lung, brain	radiation	improved	5m
Fakih et al.	46/M	T5	right	MRI	yes	2	lung, brain	radiation+cis-retinoic+*α*-interferon	improved	4m
Fakih et al.	37/F	C2	bilateral	MRI	unknown	25	lung	surgery	improved	12m
Kaya et al.	43/M	L1	left	MRI	yes	12	systemic organ metastasis	surgery	improved	6m
Altinoz et al.	43/M	T6-7	bilateral	MRI	unknown	26	lung, adrenal gland, brain	surgery	improved	>25m
Donovan et al.	41/F	C4	right	MRI	yes	0	multiple bones	surgery	progressed	6m
Asadi et al.	51/F	L1	left	MRI	no	0	brain, multiple bones	-	unknown	unknown
Parikh et al.	50/M	C5	right	MRI	yes	6	brain	radiation transformed into stereotactic radiosurgery	improved	>28m
Zakaria et al.	62/M	C7	right	MRI	yes	1	lung	surgery	improved	3m
Park et al.	44/M	T12	left	MRI	yes	6	lung	radiation transformed into surgery	improved	>8m
Gao et al.	51/M	T4-5	left	MRI	yes	72	-	surgery	improved	>3m
Present case	58/M	T12	right	MRI	yes	34	lung	surgery+radiation	improved	>6m

NE: nephrectomy; CH: chemotherapy; IL-2: interleukin-2; INF: interferon; MT: molecular target; IM: immunotherapy; PS: pulmonary surgery; BS: brain surgery; BR: brain radiation; BOA: bone radiation; AMM: antigen-modulated mini-stem cell transplant.
